# The influence of AI-driven personalized foreign language learning on college students’ mental health: a dynamic interaction among pleasure, anxiety, and self-efficacy

**DOI:** 10.3389/fpubh.2025.1642608

**Published:** 2025-09-10

**Authors:** Jun Yan, Chu Wu, Xianzhen Tan, Mao Dai

**Affiliations:** ^1^School of Foreign Languages, Yancheng Teachers University, Yancheng, China; ^2^School of Public Administration, Guangzhou University, Guangzhou, China; ^3^School of Business, Macau University of Science and Technology, Taipa, Macao SAR, China; ^4^Department of Education, Sehan University, Yeongam, Republic of Korea

**Keywords:** personalized learning, foreign language learning, mental health, pleasure, self-efficacy

## Abstract

**Introduction:**

This study examines the effects of AI-driven personalized foreign language learning on college students’ mental health, with a focus on the dynamic interaction among pleasure, anxiety, and self-efficacy. Drawing on cognitive load theory, self-determination theory, and dynamic systems theory, the research constructs a mental variable influence model to explore how emotional and cognitive factors shape learners’ experiences.

**Methods:**

A mixed-methods design was adopted, integrating questionnaire surveys, experimental research, and time series analysis. College students were randomly assigned to an experimental group receiving AI-driven personalized learning or a control group using traditional learning methods. The study tested hypotheses regarding the relationships among pleasure, anxiety, and self-efficacy and tracked their temporal evolution during the learning process.

**Results:**

Comparative analysis revealed that AI-driven personalized learning significantly enhanced pleasure, reduced anxiety, and strengthened self-efficacy compared with traditional methods. Pleasure and self-efficacy exerted a mitigating effect on anxiety, while heightened anxiety negatively influenced self-efficacy. Time series analysis further showed a phased pattern: after an adaptation period, pleasure and self-efficacy progressively increased, while anxiety levels demonstrated a sustained decline over time.

**Discussion:**

The findings provide empirical insights into the interaction mechanisms among mental variables in AI-supported learning. They suggest that AI-driven systems should integrate emotional regulation mechanisms—such as adaptive feedback, personalized emotional support, and social interaction functionalities—to enhance learners’ mental experiences and sustain learning persistence. This study contributes to the optimization of AI-driven personalized learning models, supports the design of intelligent educational technologies, and strengthens mental health protection for foreign language learners.

## 1 Introduction

The rapid advancement of artificial intelligence (AI) technology has led to its widespread application in education, with AI-driven personalized learning systems becoming integral to foreign language learning (1–3). By dynamically adjusting learning paths through real-time feedback and individualized recommendations, these systems optimize instructional content based on learners’ characteristics, progress, and preferences. While AI has significantly enhanced the efficiency and adaptability of foreign language learning, its mental implications remain insufficiently explored (4). Nowadays, foreign language teaching incorporates new methodologies that shift from traditional approaches to active methods fostering autonomy and teamwork, such as project-based learning, cooperative learning, flipped classroom, simulations, roleplays, peer tutoring. These new methodologies have a significant effect on reducing anxiety, fostering motivation and improving self-efficacy (5–7). Foreign language acquisition is inherently a complex task associated with high cognitive load, frequently accompanied by varying degrees of language anxiety, academic stress, and self-doubt, particularly at the university level where learners encounter heightened academic demands and evaluative pressures (8). Recent surveys indicate that college students often exhibit significant psychological distress in foreign language learning, including excessive worry about language tests, fear of classroom participation, and persistent learning fatigue (9). These emotional challenges not only impair learning outcomes but also potentially compromise overall mental wellbeing. Therefore, understanding the impact of AI learning systems on learners’ psychological states, specifically whether they can alleviate psychological burden while enhancing learning efficiency, represents a pressing practical concern.

AI personalized learning systems possess the potential to regulate learning stress through adaptive content recommendations, intelligent evaluation, and real-time feedback. Simultaneously, however, their highly automated feedback mechanisms, continuous task challenges, and transparent learning data may induce negative psychological experiences in some learners, such as feelings of being monitored, excessive comparison, or adaptation difficulties (10, 11). Consequently, whether AI learning systems genuinely contribute to alleviating or, conversely, exacerbating college students’ psychological burden during foreign language learning, and the underlying mechanisms, remains a notable research gap.

Within the field of psychology, pleasure, anxiety, and self-efficacy are core variables influencing learning experiences and outcomes. Pleasure, as a positive emotion, can enhance learning motivation and cognitive engagement; anxiety may disrupt information processing, leading to decreased learning efficiency; and self-efficacy directly relates to a learner’s confidence in a task and willingness to persevere. Whether AI personalized learning can simultaneously increase pleasure, mitigate anxiety, and strengthen self-efficacy, along with the internal regulatory mechanisms and dynamic relationships among these variables, are critical issues requiring in-depth exploration in current educational technology and educational psychology research.

## 2 Literature review

The application of AI in education has deepened in recent years, with personalized learning systems emerging as a crucial approach to enhance learning efficiency and improve the learning experience. AI personalized learning systems typically identify and support individual learner differences through mechanisms such as intelligent recommendations, adaptive task scheduling, and immediate feedback, thereby enhancing the precision and flexibility of foreign language learning (12, 13). In language learning, a task scenario characterized by high cognitive load, the integration of AI technology not only optimizes knowledge acquisition pathways but also subtly alters learners’ emotional experiences and psychological states. Existing research has begun to explore the impact of AI learning systems on learners’ psychological mechanisms from an interdisciplinary perspective, drawing on cognitive and emotional psychology, recognizing the significant theoretical value and applied prospects of this inquiry (14).

In AI personalized learning environments, pleasure, anxiety, and self-efficacy represent three core psychological variables frequently exhibited by learners, forming a critical nexus of cognitive-emotional-motivational interaction. Extensive existing literature confirms that these three variables are representative and highly sensitive in foreign language learning, serving as important indicators for measuring the quality of learning experience and mental health status (15). Pleasure, as a positive emotion, contributes to stimulating learning motivation and enhancing persistence and cognitive engagement. Solhi et al. (16) emotion regulation model indicated that positive emotions can increase students’ interest in tasks and autonomous participation, directly influencing learning quality. In AI learning environments, factors such as the congruence of personalized tasks and the friendliness of interactive feedback emerged as key variables affecting pleasure. Most existing studies apply AI primarily as a tool for individualized learning. However, in practice, integrating the principles of collaborative learning—such as forming heterogeneous groups composed of members with varying abilities—proves more effective in enhancing learning outcomes. Within this context, AI can function as a supportive tool within learning groups, offering intelligent assistance to facilitate heterogeneous collaboration (17).

Anxiety is one of the most widely discussed negative emotional variables in foreign language learning. Li et al. (18) theory of foreign language learning anxiety indicated that language anxiety stemmed from communication pressure, fear of evaluation, and self-cognition inconsistencies. In an AI learning environment, while the technology itself possesses the potential to alleviate anxiety, factors such as system feedback frequency, task challenge, and system transparency may also generate “technological anxiety” or “task load anxiety.” For instance, Xin and Derakhshan (19) pointed out that the high adaptability of AI systems could, for some learners, paradoxically induce feelings of uncertainty and frustration, thereby intensifying anxiety responses. Self-efficacy holds a central position in learning motivation theory, and Guo et al. (20) proposed that it is a crucial psychological mechanism influencing individual behavioral persistence, cognitive regulation, and emotional responses. In the context of foreign language learning, self-efficacy affects whether learners are willing to continuously engage, face challenges, and overcome difficulties. Lyu and Salam (21) found that AI personalized learning could enhance learners’ sense of competence through immediate feedback and personalized support, thereby improving their self-efficacy levels. Concurrently, some research indicated that for students with low self-efficacy, the complex feedback mechanisms of AI systems might lead to a loss of control, resulting in negative psychological reactions (22).

These three variables are chosen as the research focus, firstly, due to their comprehensive theoretical coverage and interactivity: pleasure represents positive emotion, anxiety represents negative emotion, and self-efficacy reflects the motivational and belief system. Secondly, these three variables frequently appear in existing foreign language learning research, have clear functional pathways, and complex interaction mechanisms, providing an analytical foundation for the emotional regulation mechanisms of AI learning systems. Compared to other single variables (e.g., learning satisfaction, stress perception), these three possess stronger theoretical integrative power and empirical operability.

Although existing literature has preliminarily explored these three variables, most studies remain confined to traditional teaching environments or focus solely on the isolated effect of a single variable. For instance, some research concentrates on AI’s role in moderating anxiety but does not simultaneously analyze positive emotional and motivational changes; other studies prioritize technical aspects, overlooking psychological feedback mechanisms. Consequently, systematically integrating pleasure, anxiety, and self-efficacy and investigating their dynamic interactive relationships within an AI learning environment represents a clear gap in current research. Furthermore, there is a present lack of longitudinal studies tracking the temporal evolution characteristics of these three variables, which also constrains the understanding of the psychological effects of AI personalized learning systems.

In summary, selecting pleasure, anxiety, and self-efficacy as the research focus is justified by solid theoretical foundations and extensive support in existing literature. This study endeavors to analyze the comprehensive impact of AI personalized learning on college students’ psychological states, starting from the dynamic interaction of these three variables. This analysis aims to provide theoretical groundwork and practical guidance for constructing intelligent learning systems that balance technical efficiency with emotional adaptability.

## 3 Research design and methods

### 3.1 Construction of the research model

The objective of this study is to examine the influence of AI-driven personalized foreign language learning on the mental health of college students, with a particular focus on the dynamic interactions between pleasure, anxiety, and self-efficacy. The selection of pleasure, anxiety, and self-efficacy as core psychological variables is grounded in robust theoretical foundations and supported by extensive empirical evidence. These three variables collectively represent key emotional and motivational dimensions in the learning process: pleasure reflects positive emotional engagement, anxiety represents a primary negative emotional experience, and self-efficacy embodies the individual’s motivational belief system. From a theoretical perspective, pleasure and anxiety correspond to the emotional dimension in the control-value theory, while self-efficacy originates from social cognitive theory. All three are integral components of emotional regulation mechanisms in learning. Moreover, these variables are highly sensitive to changes in instructional methods and learning environments, particularly in cognitively demanding contexts such as foreign language acquisition, demonstrating strong discriminatory and predictive power. Numerous studies have shown that pleasure, anxiety, and self-efficacy exert mutually influential roles in learning persistence, emotional resilience, and academic performance. To rigorously assess the mechanisms underlying these variables, a comprehensive research model is developed, drawing on the frameworks of cognitive load theory, self-determination theory, and dynamic systems theory.

Cognitive Load Theory asserts that the processing of new information by learners is contingent upon intrinsic cognitive load, extraneous cognitive load, and germane cognitive load. By optimizing these forms of cognitive load within the learning environment, the overall learning experience can be enhanced, potentially mitigating anxiety levels (23). This study posits that AI-driven personalized learning systems can modulate task difficulty, provide real-time feedback, and minimize unnecessary extraneous cognitive load, thereby influencing both learners’ anxiety and pleasure. Self-Determination Theory highlights the significance of autonomy, competence, and relatedness in fostering motivation and engagement in the learning process. AI-driven personalized learning systems have the potential to provide adaptive learning paths, intelligent content recommendations, and interactive feedback, thus reinforcing learners’ sense of competence and motivation (24–26). This study proposes that AI systems, by fostering autonomy and control, can bolster self-efficacy while concurrently alleviating anxiety stemming from external pressures. Dynamic Systems Theory underscores the dynamic, non-linear interactions of mental variables throughout the learning process, with constant fluctuations in emotional states influenced by the learners’ ongoing tasks and prior experiences (27). This study hypothesizes that AI-driven personalized learning generates diverse emotional responses, shaped not only by current learning challenges but also by past interactions. Accordingly, the research model integrates not only the direct relationships among pleasure, anxiety, and self-efficacy but also the evolving, dynamic interplay of these variables across time. Thus, the following hypotheses are proposed, as outlined in Table 1.

**Table 1:** Research hypotheses.

Hypothesis	Description
H1: AI-driven personalized foreign language learning positively influences learners’ pleasure	Adaptive learning paths and intelligent feedback features are expected to enhance interest and foster positive emotional states
H2: AI-driven personalized foreign language learning exhibits a dual effect on learners’ anxiety	AI-driven learning pathways may alleviate anxiety; however, excessively personalized challenges may heighten mental pressure
H3: AI-driven personalized foreign language learning positively influences learners’ self-efficacy	Personalized feedback and individualized pace adjustments are hypothesized to bolster learners’ confidence and self-efficacy
H4: Pleasure exerts a negative influence on anxiety	Pleasurable experiences in AI-driven environments are proposed to alleviate anxiety and enhance persistence in learning
H5: Self-efficacy exerts a negative influence on anxiety	Learners possessing higher self-efficacy are anticipated to handle challenges more effectively, thereby mitigating anxiety
H6: Self-efficacy positively influences pleasure	Increased self-efficacy is expected to correlate with heightened pleasure in the AI-driven learning environment
H7: Anxiety negatively influences self-efficacy	Elevated anxiety levels are predicted to erode learners’ confidence, thereby reducing self-efficacy

**Figure 1:**
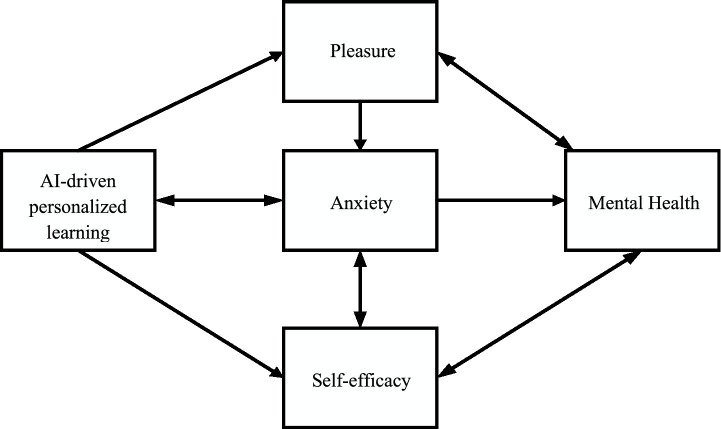
Research model of mental variables influence.

In alignment with the previously outlined hypotheses, a research model is constructed, as depicted in Figure 1.

In this model, the independent variable is AI-driven personalized learning, with pleasure, anxiety, and self-efficacy serving as mediating variables. Mental health is positioned as the dependent variable. A blend of survey techniques, experimental research, and data modeling is employed to assess the validity of the proposed hypotheses and the research model.

### 3.2 Research methodology

To rigorously assess the influence of AI-driven personalized foreign language learning on mental health in college students, a multi-method approach is utilized. This approach integrates surveys, experimental research, and data analysis to explore the intricate relationships between pleasure, anxiety, and self-efficacy. The questionnaire design is based on existing mature scales and adjusted appropriately for localization within the AI learning context. The survey instrument used in this study consists of four sections, comprising a total of 30 items, and employs a Likert 5-point scale (1 = strongly disagree, 5 = strongly agree):

1. AI-driven Personalized Learning Experience Scale (seven items): This scale assesses learners’ perceptions of the AI learning system regarding task personalization, intelligent feedback, and learning path recommendations, drawing inspiration from the Artificial Intelligence Learning Adaptability Scale (28). In its original study, this scale demonstrated good construct validity and internal consistency (Cronbach’s *α* = 0.87). For the present study, its reliability is 0.88, and minor linguistic adjustments are made after expert review to better align with the context of Chinese universities.
2. Pleasure Scale (seven items): Developed based on the academic pleasure dimension within “Control-Value Theory of Achievement Emotions,” this scale selects items related to the learning environment experience, focusing on evaluating learners’ sense of interest, satisfaction, and positive experiences when using the AI system. This scale has been widely used in multiple international studies, demonstrating strong cross-cultural adaptability (29). After review by linguistic experts and pre-testing, the scale yielded a Cronbach’s *α* coefficient of 0.89 and a Kaiser–Meyer–Olkin (KMO) value of 0.86, confirming good reliability and construct validity.
3. Anxiety Scale (eight items): This scale measures learners’ feelings of tension, unease, and task load by drawing on typical items from the Foreign Language Classroom Anxiety Scale, while also adapting them to specific anxiety sources found in AI learning environments (e.g., sense of algorithmic control, pressure from automated feedback). The adjusted version of the anxiety scale used in this study is validated to have a good one-dimensional structure, with a Cronbach’s *α* coefficient of 0.91, a KMO value of 0.88, and a significant Bartlett’s test of sphericity (*p* < 0.001), indicating its suitability for the cultural and instructional context of this study (30).
4. Self-Efficacy Scale (eight items): This scale contextualizes items from the General Self-Efficacy Scale and the self-efficacy dimension within the cognitive-motivational-self-regulatory model, making them suitable for evaluating foreign language learning tasks on AI platforms. This version has been frequently used in samples from domestic universities, demonstrating stable reliability and validity. In this study, the scale’s Cronbach’s *α* is 0.90, and its KMO value is 0.89, indicating strong measurement consistency and construct fit (31–33).

To mitigate potential social desirability and response biases associated with self-report scales, the following control measures are implemented during the study:

1. The questionnaire instructions clearly emphasize that there are no right or wrong answers, responses are completely anonymous, and data are used solely for academic research.
2. All questionnaires are completed online, with the system randomizing item order to reduce response pattern bias.
3. During data analysis, suspicious data, such as highly repetitive selections or extreme responses, are excluded to ensure sample quality.
4. Selected questionnaire results are cross-validated through subsequent supplementary interviews to enhance the credibility of data interpretation.

A total of 300 questionnaires are distributed for this study, yielding 277 responses and a response rate of 92.3%. Following data cleaning procedures, five invalid responses (e.g., those exhibiting identical answers or significant missing data) are excluded, resulting in 272 valid questionnaires, corresponding to a valid response rate of 90.7%. The participants are undergraduate students enrolled at a comprehensive university in eastern China, aged between 18 and 22. All respondents are full-time English learners with over 6 years of English education and are currently enrolled in college-level English courses, possessing a foundational proficiency in foreign language learning. To ensure sample comparability and maintain the focus of the study, students majoring in English and those with experience in advanced language proficiency tests such as IELTS or TOEFL are excluded. The sample demonstrates a balanced distribution in terms of gender, academic year, and learning background, offering strong representativeness and meeting the statistical requirements for psychological variable measurement and structural modeling in this study.

To validate the dynamic influence of AI personalized learning on learners’ psychological states, this study designs a 4-week inter-group comparative experiment. Participants are divided into two groups, one receiving foreign language learning intervention with an AI personalized learning system and the other with traditional instructional support. Both groups maintain strict consistency in learning content, study duration, task structure, and level of teacher support. The only variable differentiating the groups is the method of learning support (i.e., the presence or absence of an AI system), thereby ensuring the validity of the comparative results.

#### 3.2.1 Experimental group (AI-driven personalized learning)

This group engages in online learning using an AI personalized foreign language learning system developed by a university. The system features intelligent feedback, providing learners immediate accuracy analysis, explanations for errors, and targeted suggestions upon task completion. Additionally, the system dynamically recommends learning tasks of appropriate difficulty and type based on learners’ historical performance, enabling adaptive task recommendations. Furthermore, the system regularly adjusts learning paths based on collected learning data, generating personalized study plans for each learner, including priority review items, skills needing improvement, and task assignments (34). All learning tasks focuses on four core skills (listening, reading, speaking, and writing), ensuring consistency with the control group’s content. Each learner completes a total of 4 h of learning tasks per week (i.e., 1 h per skill). The system automatically records learning progress, which teachers can view from the backend without interfering with specific learning arrangements.

#### 3.2.2 Control group (traditional learning mode)

This group utilizes standard course materials provided by teachers for self-study. The learning content also covers the four modules of listening, speaking, reading, and writing, strictly corresponding to the task content and progress schedule of the experimental group. Materials are provided in paper or PDF format, distributed uniformly by teachers in advance. For learning feedback, the control group receives centralized feedback once a week (e.g., commentary in a WeChat group), where teachers summarize common errors but do not provide personalized guidance for individual situations. The learning path is linearly arranged and followed a fixed sequence, requiring learners to complete tasks step-by-step without an adaptive recommendation mechanism. The study time arrangement is identical to the experimental group, 4 h per week, with learning completion checked through sign-ins and periodic exercises.

To control for the influence of teacher intervention, teacher support is kept equivalent across both the experimental and control groups. The same cohort of instructors manages both groups, arranging a unified 30-min online Q&A session once per week without additional intervention. Teachers do not participate in the algorithmic adjustments of the AI system for the experimental group, nor do they provide individual tutoring for the control group, ensuring that the learning support method is the sole variable. This design aims to effectively identify the causal impact of AI personalized learning on college students’ psychological states by excluding other confounding factors.

Participants are recruited using convenience sampling from four English language courses and voluntarily participated after informed consent. For experimental intervention comparison, participants are randomly assigned to either the experimental or control group, with 136 individuals in each, ensuring balanced sample allocation. Demographically, 46.3% of participants are male and 53.7% are female, with ages ranging from 18 to 22 years and a mean age of 19.7 years. All participants possess basic English learning experience, with an average of over 6 years of study, and are currently enrolled in English courses mandated by the national English teaching syllabus at the university level. To control for the impact of background differences, English majors and students who have participated in high-level language training courses such as IELTS or TOEFL are excluded. This sample size is highly representative and meets the general sample size requirements for model building (typically *N* ≥ 200), while also satisfying the analytical needs for inter-group comparison and time-series analysis. Prior to group assignment, an equivalence test is conducted between the experimental and control groups on initial English proficiency, learning attitude, and pre-test psychological variables (pleasure, anxiety, self-efficacy). The results indicate no significant differences between the two groups (*p* > 0.05), confirming their comparability. Data collection occurred in three distinct phases, as detailed in Table 2.

**Table 2:** Data collection.

Time	Data collection
Baseline measurement (pre-experiment)	Initial levels of pleasure, anxiety, and self-efficacy were recorded through questionnaires completed by all participants
Mid-term measurement (week 2)	Using the same version of the questionnaire as in the pre-test, psychological state data were collected online from both the experimental and control groups at the end of the second week. The questionnaire measured the three core variables: pleasure, anxiety, and self-efficacy. It was completed anonymously to ensure data continuity and comparability. To maintain temporal accuracy, a unified response window was set for the two days preceding the end of the second week, and reminder notifications were sent to participants via learning management platforms such as Rain Classroom or WeCom
Final measurement (post-experiment)	A final assessment was conducted using the same questionnaire instrument as in the pre-test and mid-term assessment to evaluate the psychological states of students in both the experimental and control groups, with a focus on the overall changes in pleasure, anxiety, and self-efficacy. All participants completed the questionnaire anonymously via an online system, ensuring data continuity and comparability. During the data analysis phase, intergroup mean difference tests were employed to compare the changes in the three psychological variables before and after the intervention, aiming to determine whether the AI-driven learning model significantly improved learners’ emotional states and psychological resources over the course of the long-term intervention

## 4 Experimental analysis

### 4.1 The influence of AI-driven personalized learning on pleasure

Pleasure, as a central element of the learning process, significantly influences motivation and engagement, which, in turn, shapes learning outcomes. The AI-driven personalized foreign language learning system, through mechanisms such as intelligent feedback, tailored recommendations, and adaptive learning trajectories, aligns more closely with individual learning needs, potentially enhancing the pleasure derived from the learning experience. However, the precise pathways through which these factors exert influence require further empirical scrutiny. The study examines the pre- and post-experiment pleasure scores for both the experimental and control groups, as illustrated in Table 3.

**Table 3:** Comparison of pleasure scores before and after the experiment between the experimental and control groups.

Group	*N*	Pre-experiment *M* (SD)	Post-experiment *M* (SD)	Change
Experimental group	136	3.21 (0.75)	4.02 (0.68)	+0.81
Control group	136	3.18 (0.72)	3.42 (0.71)	+0.24

**Figure 2:**
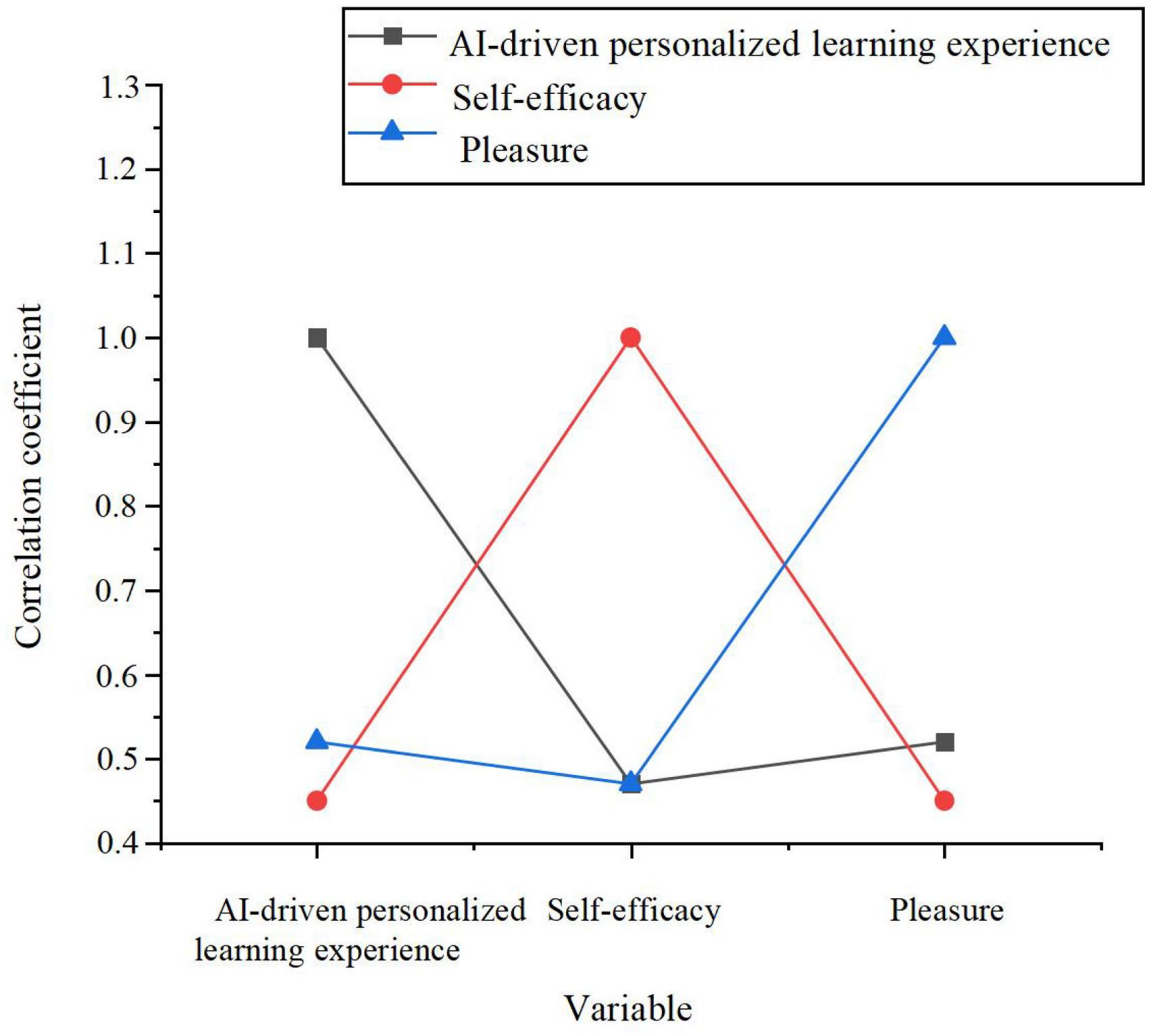
The correlation between AI-driven personalized learning experience, self-efficacy, and pleasure.

The data reveals a marked increase in the pleasure levels within the experimental group post-experiment (*M* = 4.02, *p* < 0.001), in contrast to the relatively modest improvement observed in the control group (*M* = 3.42, *p* < 0.05). This finding suggests that the AI-driven personalized learning system contributes substantially to enhancing learners’ pleasure, thereby providing preliminary evidence for H1. Additionally, the correlation coefficients between AI-driven personalized learning experience, self-efficacy, and pleasure were computed, as depicted in Figure 2.

The data presented in Figure 2 illustrates a significant positive correlation between AI-driven personalized learning experience and pleasure (*r* = 0.52, *p* < 0.01), further corroborating H1. Self-efficacy exhibits a significant positive correlation with pleasure (*r* = 0.45, *p* < 0.01), providing further validation for H6. To assess the influence of the AI learning system on pleasure and explore the moderating effect of self-efficacy, a stepwise regression analysis was performed. The results of this analysis are summarized in Table 4.

**Table 4:** Regression analysis of pleasure.

Variable	*B*	SE	*β*	*t*	*p*
AI-driven personalized learning experience	0.61	0.08	0.48	7.35	<0.001**
Self-efficacy	0.37	0.07	0.41	5.96	<0.001**
AI-driven personalized learning × self-efficacy	0.15	0.05	0.22	3.21	<0.01**

*p < 0.1, **p < 0.05, ***p < 0.01.

The analysis indicates that AI-driven personalized learning has a significant positive impact on pleasure (*β* = 0.48, *p* < 0.001), reinforcing H1. Self-efficacy also demonstrates a significant positive influence on pleasure (*β* = 0.41, *p* < 0.001), supporting H6. Furthermore, a significant interaction effect between AI-driven personalized learning and self-efficacy (*β* = 0.22, *p* < 0.01) suggests that learners with higher self-efficacy derive greater pleasure from the AI-driven learning environment.

The study, utilizing both questionnaire surveys and experimental analysis, substantiated the positive influence of AI-driven personalized learning on pleasure (H1) and confirmed the enhancement of pleasure through self-efficacy (H6). Additionally, a notable interaction effect between the AI learning system and self-efficacy emerged, suggesting that learners with higher self-efficacy tend to experience greater pleasure within the AI-driven learning environment. These findings offer valuable insights for the optimization of AI-driven foreign language learning systems. Future design efforts should prioritize strategies aimed at boosting learners’ self-efficacy, such as offering more intuitive progress feedback, personalized reward mechanisms, and other tailored approaches to further augment learners’ pleasure in the learning process.

### 4.2 Impact of AI-driven personalized learning on anxiety

Anxiety, a prevalent negative emotion in foreign language learning, may hinder learners’ focus, disrupt information processing, and negatively impact learning outcomes. AI-driven personalized learning systems, through adaptive task recommendations, real-time feedback, and intelligent assessments, offer potential to mitigate anxiety, thereby enhancing the overall learning experience. However, some studies indicate that AI systems could amplify anxiety, particularly when faced with excessive task difficulty, high feedback frequency, or a perceived lack of human-like interaction. A comparison of anxiety levels before and after the experiment between the experimental and control groups was conducted, with further analysis of the interplay between anxiety, pleasure, and self-efficacy.

**Figure 3:**
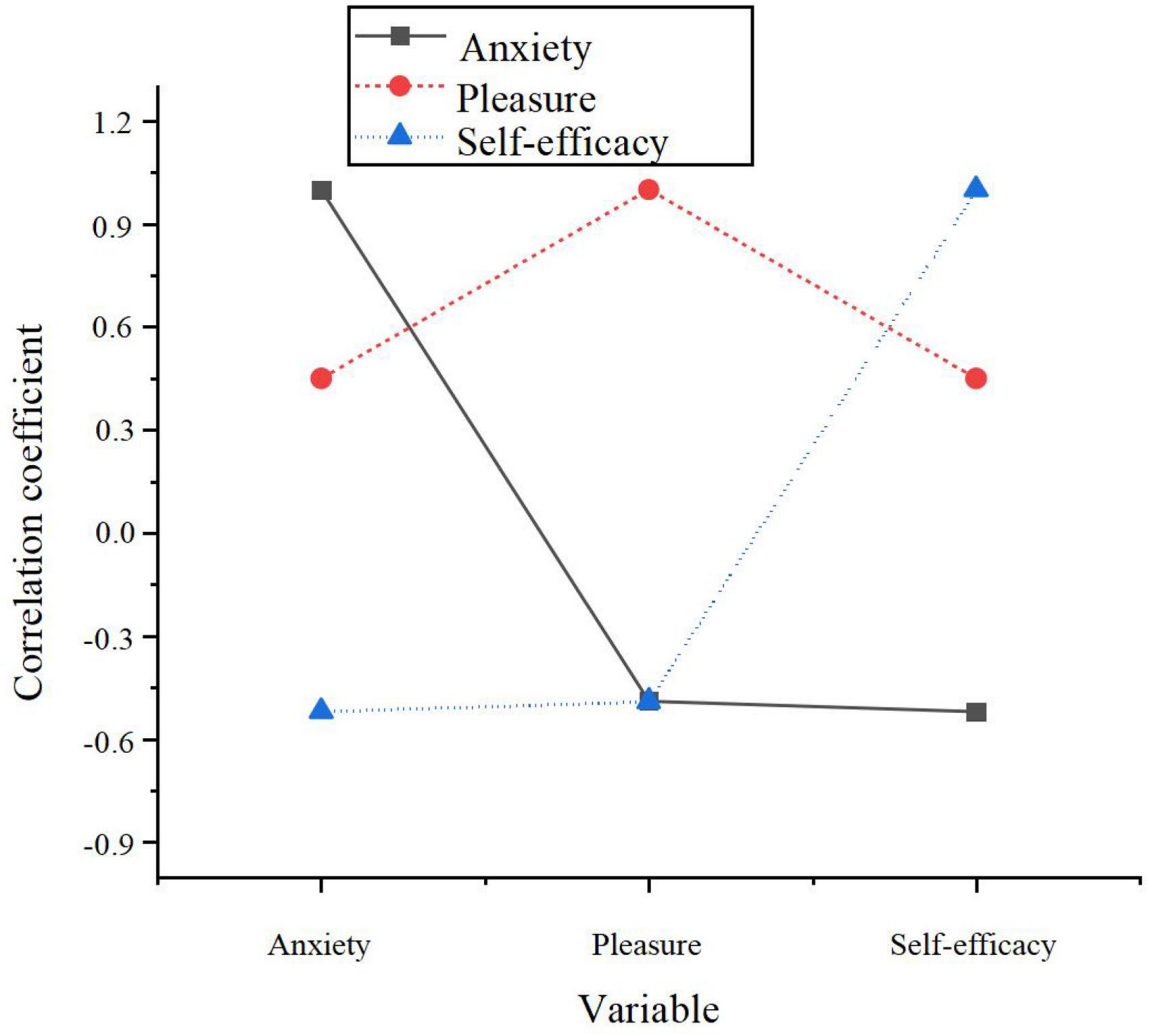
The correlation between anxiety, pleasure, and self-efficacy.

Table 5 reveals a notable decrease in anxiety levels within the experimental group post-experiment (*M* = 3.12, *p* < 0.001), suggesting that the AI-driven personalized learning system effectively mitigates learning anxiety to a considerable extent. Conversely, the reduction in anxiety within the control group was comparatively smaller (*M* = 3.49, *p* < 0.05), indicating that traditional learning approaches exert a weaker influence on alleviating anxiety. This disparity in results lends preliminary support to H2, reinforcing the significant role of AI in regulating anxiety. To further investigate the interplay between anxiety, pleasure, and self-efficacy, Pearson correlation coefficients for these three variables were computed, as depicted in Figure 3.

**Table 5:** Comparison of anxiety data before and after the experiment for experimental and control groups.

Group	*N*	Pre-experiment *M* (SD)	Post-experiment *M* (SD)	Change
Experimental group	136	3.78 (0.82)	3.12 (0.75)	−0.66
Control group	136	3.76 (0.79)	3.49 (0.80)	−0.27

A significant negative correlation between pleasure and anxiety is evident (*r* = −0.49, *p* < 0.01), corroborating H4, which posits that enhanced pleasurable experiences correspond to lower anxiety levels. Similarly, a significant negative correlation exists between self-efficacy and anxiety (*r* = −0.52, *p* < 0.01), supporting H5, and suggesting that greater self-efficacy facilitates anxiety reduction. To further explore the influence of AI-driven personalized learning on anxiety and examine the moderating roles of pleasure and self-efficacy, stepwise regression analysis was performed. The results of this analysis are presented in Table 6.

**Table 6:** Regression analysis for anxiety.

Variable	*B*	SE	*β*	*t*	*p*
AI-driven personalized learning experience	−0.58	0.09	−0.43	−6.45	<0.001**
Pleasure	−0.41	0.08	−0.36	−5.10	<0.001**
Self-efficacy	−0.47	0.07	−0.39	−5.72	<0.001**

*p < 0.1, **p < 0.05, ***p < 0.01.

As indicated in Table 6, AI-driven personalized learning exerts a significant negative impact on anxiety (*β* = −0.43, *p* < 0.001), affirming that the AI learning system contributes to reducing anxiety, thus supporting H2. Additionally, pleasure demonstrates a significant negative effect on anxiety (*β* = −0.36, *p* < 0.001), further supporting H4, highlighting that more pleasurable learning experiences lower anxiety levels. Likewise, self-efficacy exerts a significant negative effect on anxiety (*β* = −0.39, *p* < 0.001), supporting H5, and suggesting that higher self-efficacy aids in alleviating anxiety.

The study, through questionnaire surveys and experimental analysis, substantiates the role of AI-driven personalized learning in reducing learning anxiety (H2), while also validating the inhibitory effects of pleasure (H4) and self-efficacy (H5) on anxiety. In addition, the AI learning system elicits varying psychological responses among different types of learners. In particular, learners with low self-efficacy or limited technological acceptance are more prone to frustration and anxiety rebound when faced with high-difficulty tasks recommended by the system (35). This suggests that future AI-based educational design should incorporate differentiated management based on learners’ initial abilities, confidence levels, and emotional states. The challenge level of recommended tasks should be dynamically adjusted, and negative psychological impacts resulting from overly difficult tasks should be mitigated through visualized progress feedback and the reinforcement of positive emotions.

### 4.3 The impact of AI-driven personalized learning on self-efficacy

Self-efficacy refers to an individual’s judgment and belief about their capability to complete specific tasks or achieve defined goals, emphasizing task-specific expectations rather than general confidence. Unlike broader notions of confidence, self-efficacy is highly contextual and goal-oriented, playing a central role in shaping learning motivation, persistence, and outcomes. In the context of foreign language learning, learners with higher self-efficacy are generally more willing to engage actively, demonstrate stronger resilience and self-regulation when facing challenges, and are more likely to experience lower levels of anxiety. The inherent characteristics of AI-driven personalized learning systems—such as adaptive learning pathways, real-time feedback, and tailored recommendations—hold potential to bolster self-efficacy by offering individualized support that aligns with learners’ specific needs. However, variations in learners’ responses to AI-driven feedback may influence efficacy outcomes. For example, excessive feedback on errors may undermine the confidence of certain individuals. Table 7 outlines the mean self-efficacy scores, standard deviations, and observed changes for both the experimental and control groups before and after the intervention.

**Table 7:** Self-efficacy comparison for experimental and control groups before and after the intervention.

Group	*N*	Pre-experiment *M* (SD)	Post-experiment *M* (SD)	Change
Experimental group	136	3.45 (0.78)	4.12 (0.71)	+0.67
Control group	136	3.44 (0.76)	3.67 (0.75)	+0.23

**Figure 4:**
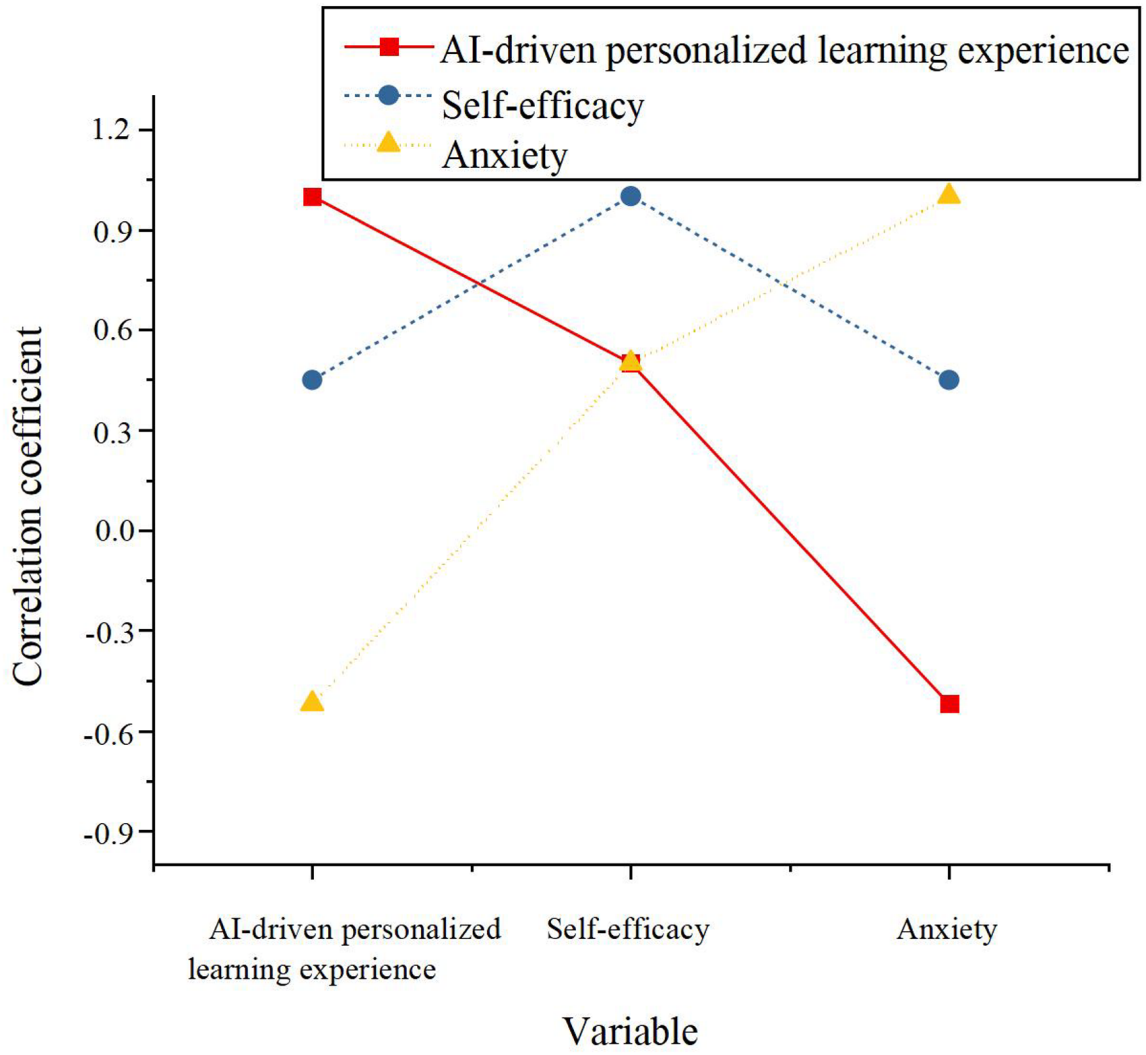
The correlation between AI-driven personalized learning experience, self-efficacy, and anxiety.

The experimental group exhibited a significant increase in self-efficacy following the intervention (*M* = 4.12, *p* < 0.001), suggesting that the AI-driven personalized learning system notably boosts learners’ confidence and perceived control over their educational journey. In contrast, the control group experienced a smaller improvement in self-efficacy (*M* = 3.67, *p* < 0.05), indicating that traditional learning methods have a more limited influence on fostering self-efficacy. This outcome provides preliminary evidence supporting H3, underscoring the positive influence of AI-driven personalized learning on self-efficacy. Subsequent correlation analysis, as presented in Figure 4, further explores the relationships between self-efficacy and other variables.

The analysis reveals a significant positive correlation between the AI-driven personalized learning experience and self-efficacy (*r* = 0.50, *p* < 0.01), offering support for H3. In contrast, anxiety exhibits a significant negative correlation with self-efficacy (*r* = −0.47, *p* < 0.01), corroborating H7, which suggests that heightened anxiety undermines learners’ confidence. To explore the influence of AI-driven personalized learning on self-efficacy further and investigate the moderating role of anxiety, stepwise regression analysis was conducted. The findings are presented in Table 8.

**Table 8:** Moderating effect regression analysis.

Variable	*B*	SE	*β*	*t*	*p*
AI-driven personalized learning experience	0.64	0.08	0.51	7.91	<0.001**
Anxiety	−0.45	0.07	−0.38	−6.24	<0.001**

*p < 0.1, **p < 0.05, ***p < 0.01.

The regression analysis confirms that AI-driven personalized learning exerts a significant positive effect on self-efficacy (*β* = 0.51, *p* < 0.001), thus further supporting H3. Conversely, anxiety demonstrates a substantial negative impact on self-efficacy (*β* = −0.38, *p* < 0.001), validating H7, and indicating that elevated anxiety levels diminish learners’ confidence.

The study substantiates the positive influence of AI-driven personalized learning on self-efficacy (H3) and the negative relationship between anxiety and self-efficacy (H7). Future AI-driven learning designs should incorporate individual differences and bolster mental adaptability mechanisms to ensure that learners with lower self-efficacy also derive benefits from personalized learning environments. These findings present critical implications for the refinement of AI-driven foreign language learning systems: forthcoming AI designs should embed mental support strategies within personalized recommendation and feedback structures, such as positive reinforcement and the display of learning accomplishments, to further elevate self-efficacy, thereby optimizing learning experiences and fostering greater learning persistence.

### 4.4 Dynamic relationship analysis of variables

In the context of foreign language learning, the interplay among pleasure, anxiety, and self-efficacy is not static but evolves dynamically. Within the AI-driven personalized learning environment, these mental factors are influenced by various elements such as personalized task recommendations, feedback mechanisms, and task difficulty adjustments. Understanding this dynamic interaction is crucial to grasping the mental impact of AI-driven learning systems. To thoroughly examine the influence of AI-driven personalized learning on mental states and validate the proposed relationships, structural equation modeling was employed. The independent variable—AI-driven personalized learning experience—encompasses features such as intelligent feedback, adaptive task recommendations, and learning path optimization. Pleasure and anxiety serve as mediating variables. AI systems may elevate learners’ pleasure through enhanced experiences, while simultaneously inducing anxiety due to the challenges posed by task difficulty. Self-efficacy, as the dependent variable, is hypothesized to be positively influenced by pleasure and negatively affected by anxiety. The path analysis results are summarized in Table 9.

**Table 9:** Path analysis results.

Path	Standardized regression coefficient (*β*)	*t*-value	*p*-value	Hypothesis verification
AI learning experience → pleasure	0.52	7.85	<0.001**	Supports H1
AI learning experience → anxiety	−0.43	−6.28	<0.001**	Supports H2
AI learning experience → self-efficacy	0.48	7.42	<0.001**	Supports H3
Pleasure → anxiety	−0.39	−5.98	<0.001**	Supports H4
Self-efficacy → anxiety	−0.36	−5.21	<0.001**	Supports H5
Self-efficacy → pleasure	0.41	6.35	<0.001**	Supports H6
Anxiety → self-efficacy	−0.40	−6.01	<0.001**	Supports H7

The findings demonstrate that AI-driven personalized learning notably enhances learners’ pleasure (H1) and mitigates anxiety (H2), highlighting the role of personalized AI features in optimizing emotional experiences during learning. The significant positive effect of AI-driven personalized learning on self-efficacy (H3) suggests that tailored learning experiences play a crucial role in fostering learners’ confidence. Pleasure exerts a substantial influence in reducing anxiety (H4), while self-efficacy similarly alleviates anxiety (H5), reinforcing the idea that positive emotional states contribute to better stress management. Additionally, self-efficacy enhances pleasure (H6), and anxiety diminishes self-efficacy (H7), illustrating the existence of a complex and interdependent regulatory system between these mental factors.

**Figure 5:**
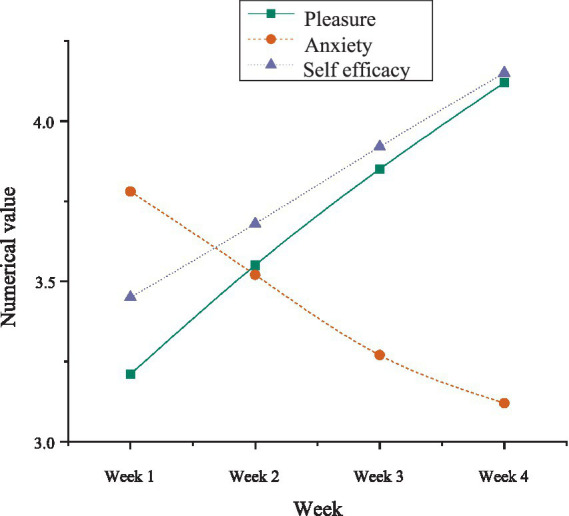
Time series analysis results.

To validate the dynamic evolution of mental variables, a time series analysis was conducted, tracking the mental states of learners in the experimental group over a span of 4 weeks and examining time-lag effects among the variables. The results, displayed in Figure 5, reveal significant patterns.

The data from Figure 5 indicate that pleasure remained relatively low during the initial week but progressively increased from the second week onward, peaking at week four (*M* = 4.12). Anxiety, initially high in the first week, steadily decreased after the learners adapted to the AI learning system, reaching its lowest point by the fourth week (*M* = 3.12). Self-efficacy showed a continuous upward trend throughout the experiment, mirroring the pattern observed for pleasure. Time-lag effects were evident in the AI-driven personalized learning system: the acceleration of pleasure growth became notably pronounced after week two, suggesting that an adaptation period was necessary for learners to fully engage with the AI model. The reduction in anxiety corresponded with the increase in both pleasure and self-efficacy, indicating that the emotional regulation effects of the AI system unfold progressively rather than instantaneously.

Through structural equation modeling and time series analysis, the study substantiated the dynamic interplay between pleasure, anxiety, and self-efficacy within the AI-driven personalized learning environment. The results reveal that AI-driven personalized learning significantly enhances pleasure, mitigates anxiety, and elevates self-efficacy, thereby affirming hypotheses H1–H3. Both pleasure and self-efficacy play critical roles in alleviating anxiety, validating hypotheses H4 and H5. Anxiety exerts a negative impact on self-efficacy, while an increase in self-efficacy further amplifies pleasure, corroborating hypotheses H6 and H7. The mental regulation effects of the AI system unfold in phases: following an adaptation period, a marked reduction in anxiety is observed, accompanied by gradual improvements in both pleasure and self-efficacy. These findings offer crucial theoretical insights for optimizing future AI learning systems, emphasizing the importance of integrating personalized learning with mental support mechanisms to enhance learners’ emotional experiences and overall learning efficacy.

## 5 Discussion

This study investigates the impact of AI-driven personalized foreign language learning on pleasure, anxiety, and self-efficacy among a sample of Chinese university students, revealing dynamic interactions among these three variables. The findings indicate that AI personalized learning systems can effectively enhance learners’ positive emotional states, alleviate anxiety, and strengthen their confidence and sense of control over learning. However, despite these positive outcomes, the findings require careful interpretation, considering the cultural context, variable control, and methodological limitations.

The results reflect the effectiveness of AI learning systems within the Chinese higher education context, particularly in university English language learning scenarios. Nevertheless, due to differences in cultural factors and educational systems (e.g., teaching methods, examination pressure, teacher roles), the generalizability of these findings across diverse cultural contexts requires further verification. For example, Western university models that emphasize autonomous learning and critical thinking might influence students’ receptiveness to AI systems’ “guided feedback.” Therefore, future research should conduct diverse comparative studies across a broader range of cultural and educational systems to confirm the universality or cultural dependency of the findings. Although the study design controls for basic demographic variables such as gender and academic year, it does not fully control for or discuss other potentially influential confounding factors that may affect psychological variables. These include participants’ prior technological experience (e.g., previous use of AI learning systems), baseline mental health levels (e.g., pre-existing long-term anxiety or emotional disorders), and foundational language proficiency (e.g., Gaokao English scores). These variables can significantly impact individual learning perceptions and emotional experiences, potentially interfering with the causal effect assessment of AI learning itself. Future research is advised to incorporate multi-level models or propensity score matching to enhance the credibility of causal inferences.

Despite this study’s initial revelation of dynamic relationships between variables and validation of hypothesizes pathways through structural equation modeling and time series analysis, causality should be interpreted with caution. As self-report scales are utilized, participants’ responses may have been influenced by social desirability bias or response tendencies. Moreover, AI learning systems, being relatively novel educational tools, may elicit positive reactions in learners over the short term due to a novelty effect or Hawthorne effect—where participants temporarily improve their learning emotions and motivation simply because they are “being observed” or “using a new tool.” Therefore, future research could integrate behavioral log data, physiological indicators, or long-term tracking studies to mitigate the limitations of self-reports.

Regarding supplementary variables like “engagement” and “motivation,” which are mentioned in the analysis, they are not included in the core path analysis of this model. However, they are occasionally referenced in the literature review and results interpretation primarily to illustrate potential mediating mechanisms through which AI systems influence psychological states. To avoid blurred variable boundaries, subsequent research should explicitly model them as independent mediating variables or completely separate them from explanations to ensure logical consistency both within and outside the model.

In conclusion, although this study provides initial evidence through its theoretical framework and empirical validation, indicating the positive potential of AI personalized learning systems in emotional regulation and psychological empowerment, its conclusions should be understood within methodological and cultural boundary conditions. This study lays the groundwork for more rigorous, cross-cultural, and longitudinal tracking studies in the future.

## 6 Conclusion

### 6.1 Research summary

This study systematically investigates the impact of AI-driven personalized foreign language learning on university students’ psychological states, focusing on the dynamic relationship among pleasure, anxiety, and self-efficacy. Through questionnaire surveys, inter-group experimental design, structural equation modeling, and time series analysis, the study finds that AI personalized learning systems demonstrate significant positive effects in three key areas:

- Enhancing learners’ pleasure.
- Significantly reducing anxiety levels.
- Strengthening self-efficacy.

Furthermore, the study reveals dynamic interactions among these variables: pleasure and self-efficacy effectively mitigate the onset of anxiety, while anxiety negatively impacts self-efficacy. These findings support the study’s seven hypotheses, validating that AI learning systems not only promote learning performance at a cognitive level but also possess a positive regulatory role at an emotional level.

### 6.2 Research limitations

Despite yielding initial theoretical insights and practical implications, this study has several limitations. The research sample is confined to Chinese university students, meaning the specificity of the cultural and educational context restricts the generalizability of the findings. Second, the experimental period is relatively short (4 weeks), which may be insufficient to capture deeper psychological change trends, especially the process of psychological adaptation after long-term AI system use. Third, this study primarily relies on self-report questionnaires, which are subject to subjective factors such as social desirability bias and response styles, and thus lacks cross-validation with behavioral data and physiological indicators. Furthermore, potential confounding variables like learning motivation, prior technological experience, and language proficiency are not fully controlled, which can impact the rigor of causal explanations.

### 6.3 Future research recommendations

Building on the limitations of this study, future research can improve in several ways:

- Expand the sample scope and conduct cross-cultural comparative studies to explore how the psychological impact of AI personalized learning systems varies across different educational backgrounds.
- Implement long-term tracking designs and phased assessment mechanisms to more comprehensively delineate the evolution trajectories of emotional variables, especially the psychological adaptation process after prolonged AI system use.
- Integrate objective data such as eye-tracking, facial expression recognition, and heart rate variability to enhance the objectivity and precision of psychological state measurement.
- Incorporate moderating variables like motivation and digital literacy to construct more complex multi-level psychological models. This would deepen the understanding of the interactive mechanisms between AI systems and learners’ psychological states, thereby providing more precise and personalized support pathways for intelligent education reform in universities.

## Data Availability

The original contributions presented in the study are included in the article/supplementary material, further inquiries can be directed to the corresponding author.
